# Biodiesel Production on Monometallic Pt, Pd, Ru, and Ag Catalysts Supported on Natural Zeolite

**DOI:** 10.3390/ma14010048

**Published:** 2020-12-24

**Authors:** Pawel Mierczynski, Magdalena Mosinska, Lukasz Szkudlarek, Karolina Chalupka, Misa Tatsuzawa, Marwa Al Maskari, Waldemar Maniukiewicz, Satriyo K. Wahono, Krasimir Vasilev, Malgorzata I. Szynkowska-Jozwik

**Affiliations:** 1 Institute of General and Ecological Chemistry, Faculty of Chemistry, Lodz University of Technology, Zeromskiego 116, 90-924 Lodz, Poland; magdalena.mosinska@dokt.p.lodz.pl (M.M.); lukasz.szkudlarek@dokt.p.lodz.pl (L.S.); karolina.chalupka@p.lodz.pl (K.C.); waldemar.maniukiewicz@p.lodz.pl (W.M.); malgorzata.szynkowska@p.lodz.pl (M.I.S.-J.); 2 Department of Chemistry, Graduate School of Science, Tokyo University of Science, Tokyo 162-8601, Japan; misatatsuzawa96@gmail.com; 3 Petroleum and Chemical Engineering Department, Sultan Qaboos University, P.O. Box 33, Muscat P.C 123, Oman; marwamaskari97@gmail.com; 4 Research Division for Natural Product Technology, Indonesian Institutes of Sciences, Jl. Jogja–Wonosari km 32, Gading, Playen, Gunungkidul, Yogyakarta 55861, Indonesia; 5 Academic Unit of STEM, University of South Australia, Mawson Lakes, Adelaide, SA 5095, Australia; krasimir.vasilev@unisa.edu.au

**Keywords:** biodiesel production, monometallic catalyst, zeolite, FAME

## Abstract

Biodiesel production from rapeseed oil and methanol via transesterification reaction facilitated by various monometallic catalyst supported on natural zeolite (NZ) was investigated. The physicochemical characteristics of the synthesized catalysts were studied by X-ray diffraction (XRD), Brunauer–Emmett–Teller method (BET), temperature-programmed-reduction in hydrogen (H_2_-TPR), temperature-programmed-desorption of ammonia (NH_3_-TPD), Scanning Electron Microscope equipped with EDX detector (SEM-EDS), and X-ray photoelectron spectroscopy (XPS) methods. The highest activity and methyl ester yields were obtained for the Pt/NZ catalyst. This catalyst showed the highest triglycerides conversion of 98.9% and fatty acids methyl esters yields of 94.6%. The activity results also confirmed the high activity of the carrier material (NZ) itself in the investigated reaction. Support material exhibited 90.5% of TG conversion and the Fatty Acid Methyl Esters yield (FAME) of 67.2%. Introduction of noble metals improves the TG conversion and FAME yield values. Increasing of the metal loading from 0.5 to 2 wt.% improves the reactivity properties of the investigated catalysts.

## 1. Introduction

The steadily growing global demand for energy, resulting in an increase in fuel consumption, is currently satisfied by the use of fossil fuels, including coal, oil and natural gas [1,2,3]. The costs of extracting fossil fuels are increasing gradually, while fuel prices fluctuate [1,4] However, it should be remembered that the world’s resources of fossil fuels are limited and gradually decreasing [5,6]. Combustion of fossil fuels leads to negative environmental effects through the emission of greenhouse gases (GHG), such as nitrogen oxides (NOx), sulfur oxides (SOx), carbon monoxide (CO), carbon dioxide (CO_2_) and other pollutants such as unburned hydrocarbons. The emission of dust generated during the combustion process is also important [7,8]. Greenhouse gases are the main reason of climate change. In addition to adverse environmental changes, emissions from burning fossil fuels are harmful to human health and some have proven carcinogenic properties [9,10]. All these issued reinforce the need for increased research efforts leading to the development of clean and renewable alternative energy sources [10]. Biodiesel is an alternative to fossil fuels due to its environmentally friendly properties such as biodegradability and absence of toxicity. In contrast to oil and other fossil fuels, biodiesel is a clean and renewable source of energy that can be produced from edible and inedible vegetable oils, animal fats, and oils from algae [11] such as rapeseed, soybean, sunflower, safflower barberry, camelina, cotton seeds, peanuts, jatropha, coconut, palm kernels, used cooking oil, tallow, and lard [12]. The world production of first-generation biodiesel is constantly growing owing to the wide range of feedstocks for the production of biodiesel via well-known and optimized commercial processes. The advantages of using biodiesel to power combustion engines compared to traditional diesel fuel are the minimum content of aromatics and sulfur in the fuel [13]. In addition, biodiesel is characterized by a higher cetane number, higher flashpoint, higher lubricity, ease of handling, transport, and storage [13]. Combustion of biodiesel produces less harmful emissions compared to the traditional diesel fuel. Due to the similar physicochemical properties to diesel oils, biodiesel is used as a biocomponent for diesel fuels [14]. Such a blend is marked with the letter B and a number indicating the percentage of biodiesel in the fuel, e.g., B2 (2% biodiesel), B5 (5% biodiesel), B20 (20% biodiesel in the blend). Moreover, biodiesel can be used in diesel engines without modification, in its pure form, designated as B100 [15,16]. The disadvantage of biodiesels are lower oxidation stability, higher pour point, higher viscosity, lower volatility and lower calorific value [13]. Structurally and chemically, biodiesel is monoalkyl esters of long-chain fatty acids, usually C_12_–C_22_ [10], formed by transesterification of triglycerides contained in oils with low molecular weight alcohols, the most commonly used alcohol is methanol; other alcohols, such as ethanol, butanol, etc., are used less frequently [10,17,18]. The most commonly obtained are methyl esters (FAME-Fatty Acid Methyl Esters) by methanolysis of triglycerodes contained in oils, in the presence of catalyst, including base/alkaline and acid catalysts, homogeneous, or heterogeneous, as well as biotechnologically produced enzymatic catalysts [18]. Industrially, biodiesel is produced by a homogeneous catalytic transesterification reactions with the use of basic alkaline catalysts (KOH, NaOH, CH_3_OK, CH_3_ONa, and CH_3_CH_2_ONa) [19]. Their wide application is due to the faster rate of the transesterification reaction compared to the acid catalysts [20,21]. Another advantage of these catalysts is the ability to catalyze the reaction at low temperature and atmospheric pressure, while providing high rate of conversion in shorter time of reaction. However, the use of strong alkaline catalysts has a number of disadvantages such as the generation of significant amounts of waste water along with the saponification of the oil and the formation of emulsions and soaps which reduce product yield and further complicate product separation. This is particularly important when the feedstock contains a high content of water and free fatty acids (more than >2% FFA). In homogeneous acid catalytic transesterification reaction mainly hydrochloric acid (HCl), sulfuric acid (H_2_SO_4_), boron trifluoride (BF3) and sulfonic acids are used as catalysts. Despite of relatively slower rate of acid-catalyzed reaction than for alkaline-catalyzed one, homogeneous acid catalysts are characterized by insensitivity of FFA and water content in the feedstock and they can simultaneously catalyze both esterification and transesterifiaction reactions [21]. These reactions can occur at mild reaction conditions with a less energy intensive. Acid-catalyzed process requires a high molar alcohol to oil ratio with a longer reaction time. Moreover, the acidic environment can cause the corrosion of reactors and pipelines, thus shortening the service life of the equipment. For both reactions, alkaline- and acid-catalyzed, the main problems are inability to reuse the catalyst or difficulties of retrieval and further separation and purification of catalyst and products [22].

Due to the numerous difficulties, obstacles, and limitations associated with homogeneous catalysts, a growing interest is focused on heterogeneous catalysts [23]. Heterogeneous catalysts have many benefits such as eco-friendly properties, safety, economy, being non-corrosive and possibility for regeneration and reuse [23]. By using these types of catalysts, the washing step of biodiesel is eliminated. Both processes of separation and purification of catalyst from products are simplified [24] resulting in high-quality glycerol characterized by high purity [25]. The advantages of heterogeneous acid catalysts are lack of sensitivity to high FFA content and moisture, ease of catalyst recovery and simultaneous esterification followed by transesterification [25]. Solid-state acid catalysts contain many centers of different strength of Brønsted or Lewis acidity. The drawback of using these type of catalysts is the necessity of using high molar ratio of alcohol to oil, high temperature, longer reaction time and/or high catalyst amount [25]. In addition, heterogeneous acid catalysts are characterized with a lower amount of acidic sites associated with leaching of catalytic active sites that can lead to product contamination and a later deactivation [25]. Synthesis of these type of catalyst is a complicated procedure, which can create additional costs (24). Examples of heterogeneous acid catalysts are heteropolyacids and their derivatives (e.g., 12-tungstophosphoric acid supported on hydrous zirconia, silica, alumina and activated carbon, H_3_PW_12_O_40_ 24 H_2_O, H_3_PMo_12_O_40_ 28 H_2_O, H_4_SiW_12_O_40_ 24 H_2_O, H_4_SiMo_12_O_40_·13 H_2_O, and H_3_PW_12_O_40_ 6H_2_O [2]), cation exchanges resins, sulfonic ion-exchange resin (e.g., EBD-100, EBD-200, EBD-300 [2]), sulfated oxides (e.g., SO_4_^2−^–ZrO_2_ [26]), sulfonic acids (e.g., benzene sulfonic acid, p-toluene sulfonic acid, 2,4-dimethylbenzene sulfonic acid [27]), acid-functionalized metal oxides, acid-functionalized carbon [28] (e.g., sulfonic acid group with biochar as the carbon support), mesoporous silica (e.g., MCM-41, MCM-48, and MCM-50). Heterogeneous base/alkaline catalysts have the same advantages as the heterogeneous acid ones—processes of catalyst separation from products and purification are simple with a high possibility of regeneration and reuse of catalyst [21]. Moreover, the reaction with these catalysts can take place under mild conditions and is less energy consuming. The disadvantages of heterogeneous base catalysts are similar to heterogeneous acid ones and consist of leaching of catalytic active sites, which may affect the reaction products by unwanted contaminants as well as the high cost of synthesis of catalyst. Unfortunately, heterogeneous base catalysts are sensitive for presence of FFA. For a feedstock with high content of FFA, formation of soap can occur leading to a decrease of biodiesel yield and possible difficulties of its purification. These catalysts should not be exposed to ambient air because of poisoning (24). Examples of heterogeneous base catalysts are alkaline earth metal oxides (e.g., CaO [28], MgO [29]), alkaline metal oxides, rare earth oxides, transitions metal oxides, alkaline doped materials [23] (e.g., Li/CaO–La_2_O_3_ [1]), alkaline ion-exchanged zeolites, alkaline ion-added zeolites, supported alkaline metal ions on alumina, silica, activated carbon; supported alkaline metal ions alkaline earth oxide (e.g., Na-K/CaO [24]); alkali metals and alkali metal hydroxides on alumina; clay materials (hydrotalcite, chrysotile, sepiolite, e.g., Mg-Fe hydrotalcites [28]), KF (Potassium fluoride) supported on alumina, lanthanide imides and nitrides on zeolites, metal nitrides, sulfides, phosphides, and carbides; organic bases grafted on microporous or mesoporous materials, anion exchanging resins (Amberlyst-A26 OH^−^) [26].

The main purpose of this work was to determine the catalytic and physicochemical properties of monometallic catalysts M/NZ (where NZ denotes natural zeolite, M = Ru, Pd, Pt, and Ag) in the transesterification reaction of rapeseed oil with methanol. Natural zeolite was chosen as the carrier for the catalyst systems. The physicochemical properties of the investigated material were investigated using a range of analytical techniques such as H_2_-TPR, NH_3_-TPD, XRD, BET, SEM-EDS, and XPS (X-ray photoelectron spectroscopy). Then, the specific physicochemical properties of the tested systems were correlated with their activity in the transesterification reaction of rapeseed oil.

## 2. Materials and Methods

### 2.1. Preparation of the Catalytic Materials

The monometallic Pt, Ru, Pd, and Ag catalysts were synthesized by impregnation method using natural zeolite samples as a support [20,27,30]. In the case of platinum, ruthenium, palladium, and silver catalysts appreciate precursor of metallic phase such us: hexachloroplatinic acid (Sigma Aldrich, Poznan, Poland), ruthenium chloride (Sigma Aldrich), palladium nitrate (Sigma Aldrich), and silver nitrate (STANLAB) were used in order to synthesize the catalytic systems. The metal loading in the case of monometallic Pt, Ru, Pd, and Ag catalysts was 0.5 or 2 wt.%, respectively. The synthesized monometallic catalysts were dried for 2 h at 120 °C, and then calcined for 4 h in an air atmosphere at 400 °C.

### 2.2. Characterization of the Catalytic Material

The catalyst surfaces morphology was investigated using S-4700 Scanning Electron Microscopy HITACHI (Tokyo, Japan), equipped with an energy dispersive spectrometer (ThermoNoran, Madison, WI, USA) (SEM-EDS). The specific surface area and pore size distribution of the catalytic materials were estimated using Surface Area and Porosity Analyzer (Micromeritics Instrument Corporation, Norcross, GA, USA) and Brunauer–Emmet–Teller (BET) method. The phase composition studies of supports and catalysts was investigated using the X-ray diffraction technique (XRD). In order to study the X-ray diffraction patterns were recorded on a PANalytical X’PertPro MPD diffractometer in Bragg–Brentano reflecting geometry (Malvern Panalytical Ltd., Malvern, UK). Cu K_α_ radiation (k = 154.05 pm) from a sealed tube was used in the 2Θ angle range 5°–90°. The reducibility of the catalytic materials was investigated by temperature programmed reduction (H_2_-TPR) technique using AMI-1 TPR system (Altamira Instruments, Pittsburgh, PA, USA). The reduction behavior of all catalytic materials was studied in the temperature range of 25–900 °C with a linear heating rate of 10 °C min^−1^. The acidity of the catalysts and supports were studied using temperature programmed desorption of ammonia (NH_3_-TPD) technique in the temperature range 100–600 °C using NH_3_ as a probe molecule and TCD detector. The monometallic catalysts before each NH_3_-TPD measurement were reduced at 300 °C, in a reducing mixture of 5% H_2_–95% Ar. The TPD-CO_2_ measurements were performed in a quartz microreactor using CO_2_ as a probe molecule. Before each test, the investigated samples were reduced, “in situ” in a mixture of 5% H_2_–95% Ar at 300 °C for 1 h. The CO_2_ was adsorbed on the catalyst surface at 50 °C for 30 min after drying in flowing Helium at 600 °C for 60 min. TPD-CO_2_ measurements were carried out in the temperature range 100–600 °C, after removing physiosorbed CO_2_ from the catalyst surface. Surface chemical composition of plasma polymerized silica nanoparticles was analyzed using a Kratos AXIS Ultra DLD spectrometer. XPS spectra were recorded with a monochromatic AlKα (hν = 1486.7 eV) radiation source conducted at an electric current of 15 mA and a voltage of 15 KeV. All binding energies were referenced to the aliphatic carbon C1s peak at 285.0 eV. The data were processed using CasaXPS (Ver. 2.3.16 Casa Software Ltd., (Casa Software Ltd., Andover, MA, USA)

### 2.3. Catalytic Activity Measurements in Transesterification of the Vegetable Oil with Methanol

The transesterification process was carried out in an autoclave using a substrate mixture of rapeseed oil and CH_3_OH with molar ratio 9/1. In all catalytic tests the weight of about 0.5 g was used. Reaction was performed at 260 °C for 2 h. Before reaction tests, catalysts were calcined at 400 °C for 4 h and reduced at 300 °C in a mixture of 5%H_2_–95% Ar. The obtained reaction products were analyzed by HPLC technique (Shimadzu). In all catalytic tests, column C-18 and as eluent was used mixture of 2-isopropanol-hexane (4/5) and methanol. The mobile phase gradient applied during each experiment was shown in Table 1. The products of the reaction were analyzed using DAD detector (wavelength: λ = 205 nm) to define of triglycerides conversion and to identification of FAME yield.

## 3. Results and Discussion

### 3.1. Transesteryfication of Vegetable Oil with Methanol Reaction

The biodiesel production via transesterification of vegetable oil with methanol reaction was carried out at 260 °C for 2 h over a range of monometallic catalysts reduced prior to use in a mixture of 5%H_2_–95%Ar at 300 °C for 1.5 h. The activation process was performed in order to improve the catalytic properties of the monometallic catalysts for the biodiesel production process. The activity of the reaction was expressed as triglycerides (TG) conversion values and fatty acids methyl esters yield. The reaction was performed in an autoclave with continuous stirring of the reaction mixture. Each process was performed for 2 h in order to obtain the final product of a mixture of the fatty acid alkyl esters, known as biodiesel. The obtained results in the studied process were presented in Table 2. The results of the activity tests clearly show that the metal loading in the catalytic systems has huge impact on the catalysts activity values in the studied process. In addition, it should be noticed that the catalytic activity test performed for calcined Pd/NZ monometallic catalysts exhibited lower TG conversion and yield of FAME production compared to the systems reduced in a mixture of 5%H_2_–95%Ar at 300 °C for 1.5 h. The catalytic activity results obtained for Pd catalyst confirmed the necessity to reduce the catalysts before the proper reaction of biodiesel production (see the results presented in Table 2).

Therefore, rest of the catalytic activity tests were done for the catalysts activated in a mixture of 5%H_2_–95%Ar at 300 °C for 1.5 h. The activity tests carried out in the biodiesel production process showed that the catalytic materials with higher metal loading exhibited higher TG conversion and yield of FAME production. The support itself (natural zeolite) is a potent catalytic material for the studied process leading to conversion of TG of 90.5% which simultaneously obtaining a high yield of methyl esters of higher fatty acids up to 67.2%. All monometallic catalysts exhibited higher TG conversion and FAME yield compared to the support itself. These results can be explained by the generation of additional active centers of metal particles which were introduced into the catalyst surface along with the appropriate precursors of the metals. The metal particles introduced onto the surface of the zeolite become active centers of the studied reaction as a result of the activation process performed in the reducing mixture. The catalytic system having the highest activity was the one containing 2% of platinum. This catalyst exhibited the highest value of TG conversion of 98.9% with the highest FAME yield. The high activity of the platinum catalysts and its high selectivity towards FAME product enable the usage of this catalytic material in transesterification process carried out from the vegetable oil and methanol mixture. The high selectivity of this catalyst makes the system ideal to use for testing it on an industrial scale. It is worth noting that so high TG conversion and FAME yield values obtained for support can be easily explained by the composition of the material. The used catalyst carrier contains in its composition Ca^2+^, Mg^2+^, K^+^, Na^+^, and Fe^3+^ ions which are widely used as a heterogenous catalysts component in transesterification reaction [31,32,33,34,35,36]. These elements are active centers in the studied reaction. Controlling the physicochemical properties through the dealumination process or the introduction of ions may increase the activity of the studied zeolite systems in various processes even in biodiesel production process. Moreover, the selectivity of the support material may be increased by the addition or activation of the system, which will be the subject of a future studies.

### 3.2. The Characterization of the Physicochemical Properties of the Investigated Catalysts

#### 3.2.1. Specific Surface Area, Porosity, and Characterization of the Natural Zeolite

Specific surface area (SSA) and porosity measurements were carried out for natural zeolite and monometallic catalysts using Micrometrics apparatus and the results are given in Table 3. The Specific surface area measurement performed for NZ showed that the support itself has 25.95 m^2^/g and pore volume equal 5.31 × 10^−2^ cm^3^/g. As is shown in Table 3, the mean pore size radius is 4.09 nm.

**Figure 1:**
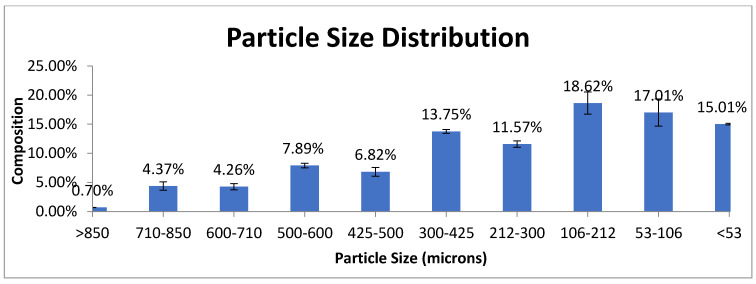
The particle size of the natural zeolite.

The natural zeolites were obtained from Gunungkidul, Yogyakarta, Indonesia, in powder with a size under 850 microns. The particle size distribution was determined by Sieve Shaker of Retsch AS 200 which is shown in Figure 1, presented below. As can be seen from the figure, the zeolite mainly contains particles with a size range of 53–300 microns (60.95%). The remaining 39.05% of the particles have size below 53 microns (15.01%) and the size between 425 and 850 microns (24.04%).

In addition to determining the SSA, the detailed characterization of the natural zeolite NZ was also done using various techniques including XPS and EDX. The ratio Si/Al in a starting material was also estimated and the results are given in Table 4.

**Figure 2:**
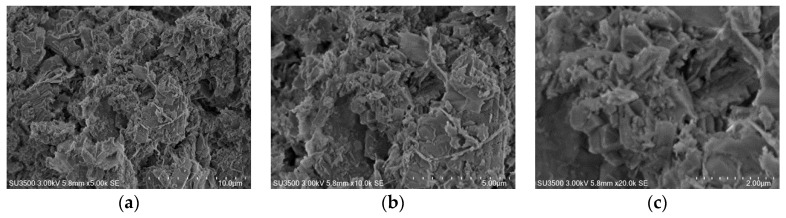
The SEM images of the natural zeolite collected from the surface with the various magnification of (**a**) 5000×, (**b**) 10,000×, and (**c**) 20,000×, respectively.

The results presented in Table 4 confirm the crystal structure of the investigated material. Natural zeolite is a mixture of calcite and mordenite-clinoptilolite structure (see XRD measurements for natural zeolite). The identified structures included in the natural zeolite composition confirmed the occurrence of Ca, Mg, Si, Al, Na, O, Fe, and K elements. It is also worth mentioning that the composition of this material can explain the reductive properties (see reduction part of this work) of the catalytic material and its structure. The structure morphology of the natural zeolite was also studied by scanning electron microscopy and the collected images from the surface is shown in Figure 2. The presented images were done at different magnifications.

The SSA results clearly shows that all of the investigated catalysts and support are characterized by SSA in the range 16.11–26.37 m^2^/g. The highest SSA value equal 26.37 m^2^/g was obtained for 2%Ru/NZ catalyst. The lowest size of specific surface area exhibited 2%Pt/NZ catalyst. The same tendency was observed in the case of the monolayer capacity. In all cases the values calculated for the catalytic materials exhibited practically the same values. Only in the case of the platinum catalyst, the specific surface area and the average pore size values differed from other systems. These results confirmed that the in the case of the monometallic catalysts containing 2%wt. of Pt specific surface area is not a critical parameter which significantly affects the catalytic reactivity of catalytic systems during the biodiesel production process.

#### 3.2.2. The Acidity Properties of the Synthesized Catalysts Systems

The acidity properties of the catalytic materials were extensively studied by NH_3_-TPD method and the results are given in Table 5. Ammonia was used as a probe molecule to determine the quantity of the acidic centers present on the catalyst surface and their division into weak, medium, and strong power centers. The ammonia is an ideal molecule to study the electron-acceptor properties of the investigated material. The free pair of electrons present on the nitrogen atom allow to estimate the acidic properties of the catalytic material. All investigated materials exhibited three types of acid centers on the catalyst surfaces in the temperature range of 100–600 °C (weak centers—100–300 °C, medium centers—300–450 °C, strong acid cites—450–600 °C). The lowest total acidity value of 1.0 mmol/g showed the NZ. In the case of the 0.5%Ag and 0.5%Pd catalysts supported on NZ, the total acidity was equal to 1.2 mmol/g. The NH_3_-TPD results obtained for the rest of the catalytic materials showed that systems exhibited practically the same total acidity in the range 1.9–2.3 mmol·g^−1^. Whereas the quantity of strong acid centers was the same for all catalysts. The greatest differences were in the quantity of weak and medium acidic centers. In the case of medium power centers, the highest quantity of these centers exhibited 2%Pd/NZ, 2%Pt/NZ, and 2%Ru/NZ. These results may explain the differences in catalytic activity of the evaluated materials. It should be noted that for these systems we observed the highest values of TG conversions in transesterification reaction.

#### 3.2.3. Basic Properties of the Synthesized Catalyst Systems

CO_2_-TPD experiments were used to characterize catalytic materials alkalinity and the results are shown in Table 6. The results show that all investigated monometallic catalysts systems exhibited three desorption centers with different strength (weak, medium, and strong). The total basicity values of all materials shows that the most alkalinity material was NZ (0.8 mmol/g) itself which showed two types of alkaline sites on the surface, namely, low and strong basic sites. The obtained results gave evidence that carrier system desorbs the highest amount of CO_2_ in the temperature range 100–600 °C compared to the monometallic catalysts. The CO_2_-TPD measurements presented in the Table 6 shows that support and all monometallic catalysts excluding silver catalyst exhibited the same quantity of CO_2_ desorbed from the weak centers. These results confirmed that the alkalinity of the studied systems does not explain the differences in the observed values of triglyceride conversion and the yield of the formed methyl esters of higher fatty acids.

#### 3.2.4. Phase Composition Studies of Monometallic Catalysts

**Figure 3:**
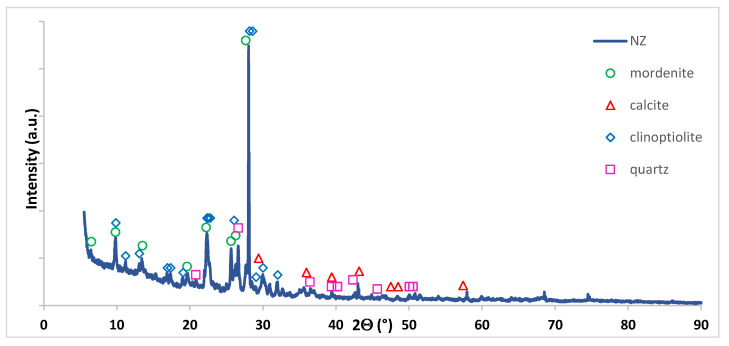
XRD diffraction curve of the natural zeolite.

The XRD analysis was performed for the carrier material itself and the analysis of these measurements were given in Table 6 and in Figure 3. As we can easily see from the obtained results, natural zeolite structure contains calcite, mordenite, clinoptilolite (zeolite) and quartz. The obtained diffraction curve for NZ with the identified crystal structures are presented in Figure 3.

**Figure 4:**
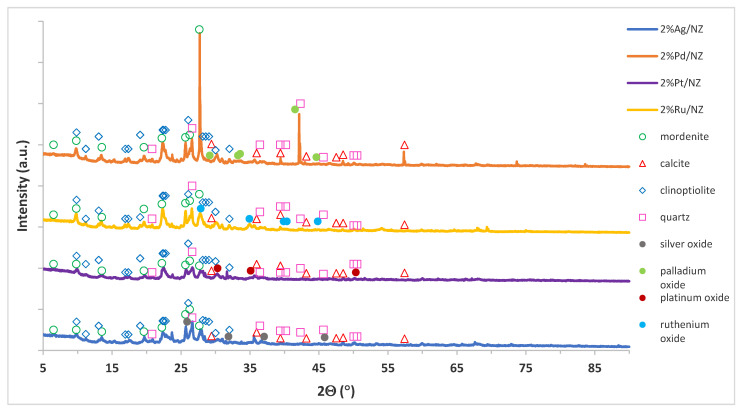
XRD diffraction curves of the calcined monometallic 2%M/NZ (where M = Ag, Pd, Pt, Ru) catalysts in an air atmosphere at 400 °C for 4 h.

XRD technique was also used to determine the phase composition studies of the monometallic supported catalysts after their calcination process. An analysis of the crystal structure of the support system (NZ) is presented in the previous section concerned with the determination of the specific surface area of the prepared catalysts. In the case of the monometallic systems the phase composition of the investigated catalysts is given in Figure 4. In the case of monometallic catalysts, the crystalline structures derived from the support (calcite, mordenite, and clinoptilolite) and metal oxide phases depending on the type of catalyst were observed on the diffraction curves.

Analyses of the diffraction patterns of the calcined monometallic catalysts showed the respective diffraction peaks for the relevant catalysts. In the case of the silver catalyst the diffraction peaks attributed to silver oxide (Ag_2_O) were visible at 2 theta 25.88°, 31.88°, 37.05°, and 45.83°. Other diffraction peaks positioned at 2θ = 27.86°, 34.91°, 39.88°, 40.35°, and 44.8° assigned to ruthenium oxide (RuO_2_) were detected for the 2%Ru/NZ catalyst. In the case of the platinum and palladium supported catalysts, diffraction peaks located at 2θ = 30.30°, 35.10° and 50.35° for PtO_2_ and at 2θ = 29.17°, 33.26°, 33.57°, 41.53°, and 44.63° for PdO were observed. The rest of the diffraction peaks visible on the XRD patterns originated from the crystallographic phases forming the support system, namely calcite, mordenite, clinoptilolite, and quartz phases.

#### 3.2.5. Reduction Behavior of the Catalytic Materials

**Figure 5:**
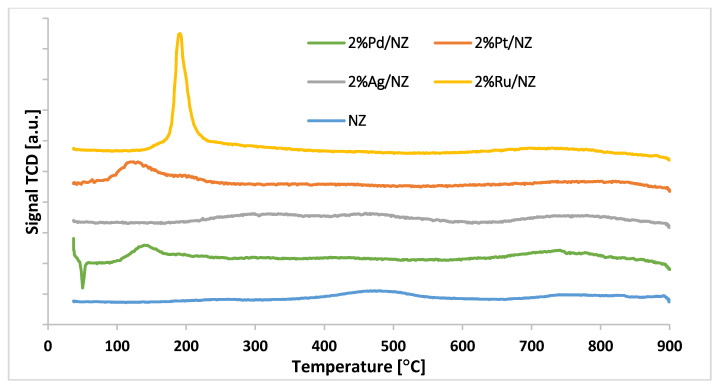
H_2_-TPR curves recorded for NZ and various monometallic catalysts M/NZ (where M = Ag, Pd, Pt, Ru) calcined in an air atmosphere at 400 °C for 4 h.

The reduction behavior of NZ and monometallic catalysts was also studied in this work. The results of the H_2_-TPR- measurements are given in Figure 5. The TPR curve of Natural zeolite shows three reduction stages. These reduction stages are attributed to the reduction of iron oxides. The first two reduction peaks are assigned to the reduction of hematite to magnetite and magnetite to wüstite (FeO) [37,38].

**Figure 6:**
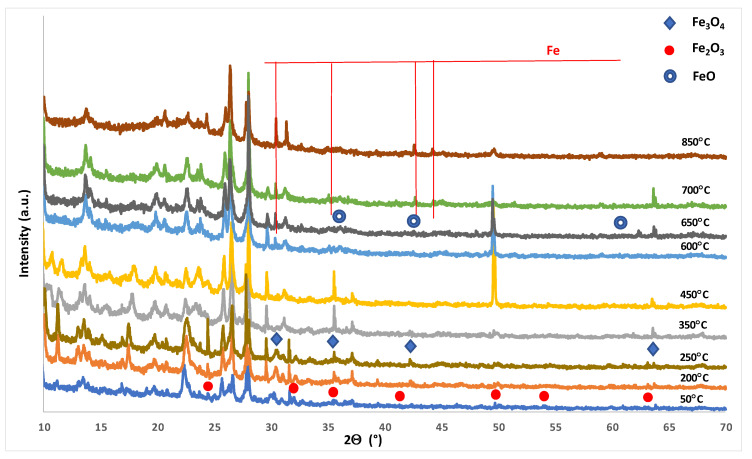
XRD, “in situ” reduction measurements performed in a mixture of 5%H_2_–95%Ar for pristine NZ material.

The high temperature reduction stage with the maximum of hydrogen consumption peak in the temperature range 650–900 °C visible on the H_2_-TPR curve is assigned to the reduction of FeO to the metallic iron (Fe^0^). In order to confirm the reduction studies performed for NZ material reduction, “in situ” studies using XRD technique was done. The results of the reduction studies of the NZ material were presented in Figure 6. Selected XRD curves are shown in Figure 6 to better visualize the progress of the reduction process. In addition, on the selected XRD patterns only reflexes assigned to the reduction process of hematite were marked. As we can see on the diffraction curves recorded for natural zeolite in the temperature range 50–200 °C we can distinguish the reflexes assigned to mordenite, clinoptilolite, quartz, calcite, and hematite phases. During the increasing of the reduction temperature to 250 °C the following reduction process is observed, confirmed by the appearance of a magnetite phase coming from the reduction of hematite [37]. The magnetite phase was visible on the diffraction patterns recorded for the investigated material in temperature range 250–600 °C. In this temperature range the calcium oxide phase formed from the decomposition of the calcite phase was also detected. The XRD pattern recorded for the studied system at 650 °C shows the reflexes attributed to mordenite, clinoptilolite, quartz, calcium oxide, wüstite, and metallic iron phases. It is worth noticing that the appearance of the wüstite phase is connected with the reduction process of magnetite phase [38]. Further increasing of the reduction temperature result in disappearance of wüstite phase, what is explained by the reduction process. Diffraction curve of NZ material recorded at 850 °C confirmed the presence of metallic iron, mordenite, clinoptilolite, and quartz phases.

In the case of all studied monometallic supported catalysts the remaining reduction stages connected with the iron oxide species reduction were also visible on the TPR profiles of other catalytic systems. In addition, in the case of palladium, platinum, ruthenium and silver catalysts, additional metal oxides phases were reduced in the low temperature range. The H_2_-TPR profile of the Pd catalyst shows two effects at low temperature. The first evolution of hydrogen effect is assigned to the decomposition of β-PdHx phase. This hydride phase can decompose when the temperature increases above 70 °C and released molecular hydrogen generates a desorption peak on the TPR profile [39,40]. The second reduction stage with the maximum of hydrogen consumption peak is attributed to the reduction of PdO to metallic Pd [41,42]. The reduction profile of 2%Ag/NZ catalyst showed the reduction peaks assigned to the reduction of the same oxidic species which were observe in the case of the carrier itself as well as the effect assigned to the reduction of Ag_2_O. A low temperature range of 100–200 °C, the reduction peak assigned to the reduction of Ag_2_O can be easily observed. Published studies [43,44] reported the same reduction peaks connected with the reduction of silver oxide to metallic silver. The reduction curve of 2%Pt/NZ catalyst shows reduction effects attributed to the reduction of the carrier itself and PtO_2_ through PtO to metallic Pt. The same result was observed also by other authors [42,45]. The reduction profile of 2%Ru/NZ catalyst shows the reduction stages assigned to the reduction of iron oxide species and RuO_2_ to metallic Ru through RuO. The same reduction behavior of ruthenium oxide species was also observed by other authors [46]. The reduction properties of ruthenium catalysts was also studied in reference [41]. The authors reported two partially resolved hydrogen consumption peaks observed in the TPR profile recorded for the investigated catalyst. The first reduction peak was attributed to the reduction of RuO_2_ to RuO, While the second reduction effect was assigned to the reduction of RuO to metallic Ru. Wang et al. [47] also reported the reduction of Ru^4+^ to metallic ruthenium Ru^0^. Gao et al. [48] also studied the Ru containing catalysts and they assigned the observed reduction stages to well-dispersed RuO_x_ and RuO_x_ with strong interaction with support surface species.

#### 3.2.6. The Morphology of the Investigated Catalysts

**Figure 7:**
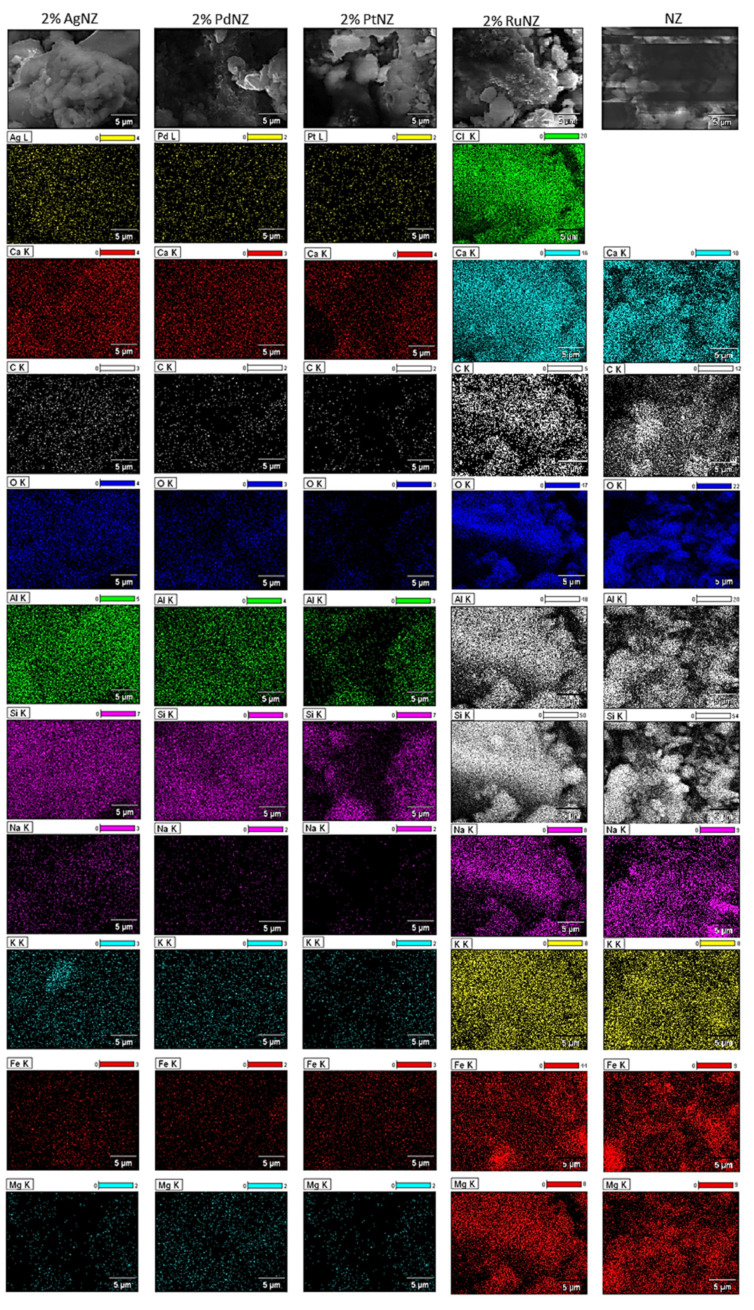
SEM-EDS images of the calcined support NZ and monometallic M/NZ catalysts in an air atmosphere at 400 °C for 4 h.

SEM-EDS measurements were also carried out for support and monometallic catalysts. Scanning electron microscopy is a useful surface technique to determine the morphology and composition of the surface of the catalytic material. SEM images collected from the surface of the investigated catalytic materials were given in Figure 7. The presented SEM and EDS images of the support and monometallic catalysts confirms the composition of the investigated material. The analysis of the support itself shows the presence of the elements such as Mg, Ca, K, Fe, Si, Na, Al, and O on the carrier surface. Analogical SEM-EDS measurements were also performed for monometallic supported catalysts and the results are given on the same Figure 6. The analysis of the monometallic catalyst surface showed that all catalysts contain the same elements which were detected for support material. In addition, in the case of the platinum, palladium and silver catalysts the presence of appropriate noble metals, such as Pt, Pd, and Ag, on the surface of the catalysts were confirmed for the tested systems. In addition, in the case of ruthenium catalyst the presence of Cl on the surface was also detected. The lack of Ru on the surface of Pt/NZ catalyst is due to the fact that the radiation energy values characteristic for ruthenium and chlorine are almost identical, which makes it difficult to identify these elements in the same sample. The signals come from Cl and Ru overlap each other’s on EDS spectra, what can explain the lack of this element on the SEM-EDS spectra. The presence of this element was confirmed by XRD method in the case of the calcined supported catalyst. In addition, SEM-EDS results gave evidence that the dispersion of precious metals and other elements on the surface is not uniform. The surface analysis showed that there are regions with a higher metal content on the support surface, which is typical for catalysts prepared using the wet impregnation method.

## 4. Conclusions

In summary, monometallic catalysts supported on natural zeolite were prepared by an impregnation method and all materials, including the support were tested in biodiesel production reaction in order to select the best catalytic system. The physicochemical properties of the catalysts were also correlated with the reactivity results in a biodiesel production process. The physicochemical properties were tested by H_2_-TPR-, NH_3_-TPD, BET, XRD, SEM-EDS, and XPS techniques. The activity results clearly show that the reactivity of the studied systems in transesterification of rapeseed oil strongly depend on the type of monometallic catalyst and the metal loading in the catalytic material. It was proven that the metal loading in the catalytic systems has huge impact on the catalysts activity values. It was also confirmed that the introduction of the noble metal (2% wt. of metal) into the surface of the natural zeolite improve the yield of the obtained methyl esters and the TG conversion value of support material. The most active system in transesterification reaction was platinum catalyst which showed the highest value of triglyceride conversion and the efficiency of the methyl esters of higher fatty acids, which can be explained by its the high alkalinity and easy reducibility compared to the other monometallic catalysts. In addition, the reactivity results showed that support itself exhibited high activity in transesterification of rapeseed oil. The conversion of TG was 90.5% in the case of the reaction performed over NZ material and relatively high yield of FAME of 67.2%. The obtained results in this work confirmed that such the reported systems could be a potent catalytic material to be used in industrial processes in biodiesel production technology.

## Figures and Tables

**Table 1:** Phase gradient used in the HPLC measurements.

Mobile Phase Gradient.			Flow Rate mL·min^−1^
Time (min)	Solvent A (%)	Solvent B (%)	
0.0	100	0	1
20.0	100	0	1
45.0	0	100	1
70.0	0	100	1
75	100	0	1

Solvent A: Methanol; Solvent B: 2-Propanol/Hexane = 4/5; Injection Volume: 1 Μl; Column Temperature: 25 °C.

**Table 2:** The results of the transesterification process performed over various monometallic catalysts activated in a mixture of 5%H_2_–95% Ar at 300 °C for 1.5 h, calcined system, or pristine natural zeolite (NZ) material.

Catalyst	Reaction Temperature (°C)	Reaction Time (h)	Molar Ratio Oil: Methanol	Calcination Temperature (°C)	Catalyst Weight (g)	Triglycerides Conversion (%)	FAME Yield (%)
NZ	260	2	1:9	400	0.5	90.5	67.2
0.5%Ru/NZ	260	2	1:9	400	0.5	90.2	48.0
0.5%Pd/NZ	260	2	1:9	400	0.5	79.9	62.4
0.5%Pt/NZ	260	2	1:9	400	0.5	89.6	47.4
0.5%Ag/NZ	260	2	1:9	400	0.5	81.0	47.9
2%Pt/NZ	260	2	1:9	400	0.5	98.9	94.6
2%Pd/NZ	260	2	1:9	400	0.5	98.9	73.8
2%Pd/NZ (cal.)	260	2	1:9	400	0.5	94.6	64.8
2%Ru/NZ	260	2	1:9	400	0.5	95.3	71.0
2%Ag/NZ	260	2	1:9	400	0.5	96.3	71.6

**Table 3:** The specific surface area measurements calculated for the synthesized catalytic materials calcined at 400 °C for 4 h in an air atmosphere.

Material	BET Surface Area (m^2^/g)	Monolayer Capacity (cm^3^/g)	Average Pore Radius (nm)
**NZ**	25.95	-	4.09
**2%Ag/NZ**	21.32	0.076	6.20
**2%Pd/NZ**	21.35	0.076	6.38
**2%Pt/NZ**	16.11	0.067	6.67
**2%Ru/NZ**	26.37	0.075	5.84

**Table 4:** Element composition and Si/Al Ratio of the natural zeolite (XPS, EDX).

Element	EDX	XPS
Ca	3.55	6.98
C	-	7.61
O	52.02	55.38
Al	7.95	5.84
Si	31.05	19.27
Na	1.75	-
K	1.12	-
Fe	2.55	4.42
Mg	-	0.51
SiO_2_/Al_2_O_3_	4.42	3.85

**Table 5:** The amount of NH_3_ adsorbed on support surface (calcined in an air atmosphere for 4 h at 400 °C), monometallic reduced catalysts (reduction at 300 °C in a mixture of 5% H_2_–95% Ar mixture), calculated from the NH_3_- temperature programmed reduction (TPD) profiles.

Catalytic Systems	Total Acidity (mmol/g) 100–600 °C	Weak Centers (mmol/g) 100–300 °C	Medium Centers (mmol/g) 300–450 °C	Strong Centers (mmol/g) 450–600 °C
NZ	1.0	0.6	0.3	0.1
0.5%Ag/NZ	1.2	0.7	0.5	0.1
2%Ag/NZ	1.9	0.7	0.8	0.3
0.5%Pt/NZ	2.0	1.1	0.7	0.1
2%Pt/NZ	2.1	1.2	0.7	0.2
0.5%Pd/NZ	1.2	0.6	0.5	0.1
2%Pd/NZ	1.9	0.9	0.9	0.1
0.5%Ru/NZ	2.0	1.0	0.6	0.2
2%Ru/NZ	2.3	1.3	0.9	0.1

**Table 6:** The amount of CO_2_ adsorbed on support surface (calcined in an air atmosphere for 4 h at 400 °C), monometallic reduced catalysts (reduction at 300 °C in a mixture of 5% H_2–_95% Ar mixture), calculated from the CO_2_-TPD profiles.

Catalytic Systems	Total Basicity (mmol/g) 100–600 °C	Weak Centers (mmol/g) 100–300 °C	Medium Centers (mmol/g) 300–450 °C	Strong Centers (mmol/g) 450–600 °C
NZ	0.8	0.4	0.0	0.4
2%Ag/NZ	0.5	0.3	0.1	0.1
2%Pt/NZ	0.6	0.4	0.1	0.1
2%Pd/NZ	0.5	0.4	0.05	0.05
2%Ru/NZ	0.6	0.4	0.1	0.1
